# Guillain-Barré Syndrome Associated with COVID-19 Vaccination

**DOI:** 10.3201/eid2805.212145

**Published:** 2022-05

**Authors:** Josef Finsterer, Fulvio A. Scorza, Carla A. Scorza

**Affiliations:** Neurology and Neurophysiology Center, Vienna, Austria (J. Finsterer);; Disciplina de Neurociência. Universidade Federal de São Paulo/Escola Paulista de Medicina, São Paulo, Brazil (J. Finsterer, F.A. Scorza, C.A. Scorza)

**Keywords:** COVID-19, coronavirus disease, SARS-CoV-2, severe acute respiratory syndrome coronavirus 2, viruses, respiratory infections, zoonoses, vaccine-preventable diseases, vaccination, immune-mediated, Guillain-​Barré syndrome, Taiwan

**To the Editor:** With interest we read the article by Shao et al. (*1*) about the frequency of severe acute respiratory syndrome coronavirus 2 (SARS-CoV-2) vaccination–associated Guillain-​Barré syndrome (SCoVaG) among 18,269 healthcare workers in Taiwan who had received the AstraZeneca vaccine (AZV; https://www.astrazeneca.com). Only 1 vaccinee experienced SCoVaG during the study period (*1*). The study is appealing but raises concerns.

Recently, our review of 19 SCoVaG patients, for whom data were collected through June 2021, was published (*2*). The 9 men and 10 women in the study were 20–86 years of age. All patients experienced SCoVaG after the first vaccine dose. AZV was given to 14 patients, the Pfizer-BioNTech (https://www.pfizer.com) vaccine to 4 patients, and the Johnson & Johnson (https://www.jnj.com) vaccine to 1 patient. Latency between vaccination and SCoVaG onset ranged from 3 hours to 39 days. Patients received intravenous immune globulin (n = 13), steroids (n = 3), or no therapy (n = 3). Six patients required mechanical ventilation. One patient recovered completely; 9 achieved partial recovery (*2*). Only 1 of the studies included in our review mentioned the total number of vaccinated persons (*3*); in that study, 7 persons among 1.2 million vaccinated persons were found to have SCoVaG (*3*).

In addition, data on 389 patients with SCoVaG were collected in a recent review about the neurologic adverse events of SARS-CoV-2 vaccination (*4*). However, no individual data were provided for 337 of these patients (*4*). Among the 53 patients for whom individual data were available, AZV was given to 39 patients, Pfizer-BioNTech vaccine to 9 patients, and Johnson & Johnson vaccine to 2 patients.

For the Shao et al. report (*1*), we wondered why the oldest healthcare worker was 86 years of age. Also missing were the specific treatment and outcome of the patient with SCoVaG.

Available data suggest that SCoVaG is a rare complication of SARS-CoV-2 vaccination, irrespective of the vaccine brand used. SCoVaG should be diagnosed early so treatment can be initiated promptly. Whether the beneficial effect of SARS-CoV-2 vaccination outweighs the risk for adverse events (e.g., Guillain-​Barré syndrome) remains a matter of discussion (*5*).
